# ﻿New records of Nitidulidae (Nitidulidae, Coleoptera) species in Canada, Ontario, and Manitoba

**DOI:** 10.3897/zookeys.1156.94589

**Published:** 2023-03-24

**Authors:** Sharon E. Reed, David Dutkiewicz, Fiona Ross, Jennifer Llewellyn, Hannah Fraser

**Affiliations:** 1 Ontario Forest Research Institute, Ministry of Natural Resources, and Forestry, 1235 Queen Street, Sault Ste. Marie, Canada Ministry of Natural Resources, and Forestry Sault Ste. Marie Canada; 2 Invasive Species Centre, Sault Ste Marie, Canada Invasive Species Centre Sault Ste Marie Canada; 3 Manitoba Natural Resources and Northern Development, Winnipeg, Canada Manitoba Natural Resources and Northern Development Winnipeg Canada; 4 Ontario Ministry of Agriculture, Food, and Rural Affairs, Guelph, Canada Ontario Ministry of Agriculture, Food, and Rural Affairs Guelph Canada

**Keywords:** *Bretziellafagacearum*, *Carpophilus*, *Cychramus*, *Glischrochilus*, oak wilt, *Stelidota*

## Abstract

Nitidulidae trapping performed from 2018 to 2021 to characterize flight behaviors of potential vectors of the oak wilt pathogen yielded three new species records for Canada, six new species records for Ontario, and three new species records for Manitoba. The new records for Canada include Carpophilus (Ecnomorphus) corticinus reported from Ontario, C. (Myothorax) nepos reported from Ontario and Manitoba, and Glischrochilus (Librodor) obtusus reported from Ontario. In addition, the following species are first recorded in Ontario: Carpophilus (Ecnomorphus) antiquus, C. (Megacarpolus) sayi, *Stelidotacoenosa*; and also in Manitoba: Carpophilus (Megacarpolus) lugubris and *Cychramusadustus*. Collection data is provided for the two provinces and national records.

## ﻿Introduction

The family Nitidulidae occurs globally with at least 4,500 species, of which, 173 species are found in North America (Habeck 2002; Marske and Ivie 2003). Also known as sap beetles or picnic beetles, Nitidulidae feed on flowers, fruits, fungi, stored products, decaying and fermenting plant tissues, carrion, and other insects. Feeding on fermenting sugars of fruits and vegetation is most common. Feeding by the beetles introduces fungi and bacteria to damaged plant tissues, resulting in further decay and fermentation. This appetite attracts the beetles of several Nitidulidae genera to the fruit-like smell of fungal mats produced by the oak wilt pathogen, *Bretziellafagacearum* (T.W. Bretz) J Hunt, which is an invasive species of fungus affecting oak trees (*Quercus* spp.) (Downie and Arnett 1996). Nitidulids become contaminated with fungal spores when they visit oak wilt mats. New infections occur when contaminated beetles leave the oak wilt mats and fly to fresh wounds on healthy oak trees, spreading the disease across the landscape (Jagemann et al. 2018). Identifying beetle species associated with movement of the fungus is important for establishing management criteria.

Oak wilt has been killing oak trees in the United States for more than half a century, spreading to 24 states and residing within 1 km of Canada on Belle Island, Michigan (Juzwik et al. 2008; USDA-FS 2019). Oak trees are susceptible to this fungus with some species dying within weeks of the initial infection. Oak wilt can infect neighboring trees through root-to-root contact (Bruhn et al. 1991), however transmission by nitidulids is considered more concerning because of the potential for transmission over greater distances and the formation of many new infection epicenters (DiGirolomo et al. 2020).

Overland spread of oak wilt by nitidulid beetles can be reduced by avoiding oak wounding when nitidulids are most active. A multi-year study of Nitidulidae beetle flight behavior was carried out at 21 localities in the following three Canadian provinces, Manitoba, Ontario, and New Brunswick. The study also aimed to describe the diversity of nitidulids that could be involved in oak wilt transmission. Beetles were collected using flight interruption traps. In total, there were 49 nitidulid species collected across the three provinces with three new records in Manitoba, six new records in Ontario, and three new records for Canada from Ontario and Manitoba. No new records are reported for New Brunswick.

## ﻿Materials and methods

**Figure 1.**
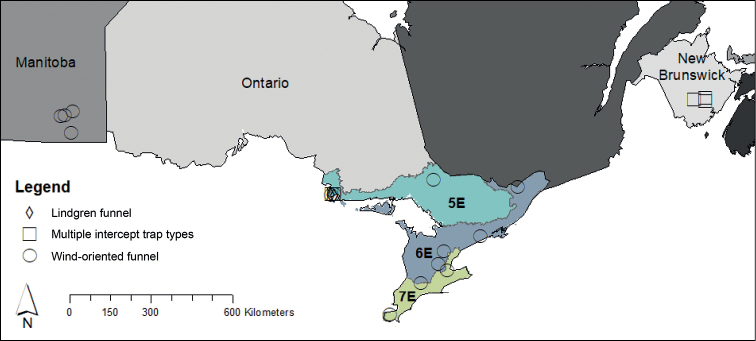
Geographic localities in Canada and Ontario ecoregions where flight intercept traps were used to collect Nitidulidae. Abbreviations: 5E – ecoregion 5, Georgina Bay.; 6E – ecoregion 6, Lake Simcoe-Rideau; 7E – ecoregion 7, Lake Erie-Lake Ontario.

Traps were deployed at 14 localities in Ontario, four in Manitoba, and three in New Brunswick (Fig. 1). Flight intercept traps were installed in Manitoba in or near Winnipeg. These four localities are in the Interlake Plain and Lake Manitoba ecoregions (Smith et al. 1998). Both ecoregions experience short, warm summers and long, cold winters with slightly warmer temperatures in the Lake Manitoba Ecoregion. Deciduous and coniferous forest remain but large areas have been cleared for agriculture. Localities included an airport and three provincial parks. Bur oak (*Quercusmacrocarpa* Michx.) is the only oak species that occurs in these ecoregions (Farrar 1995).

All New Brunswick localities were in the Grand Lake Lowlands (Fig. 1; Province of New Brunswick 2007). This ecoregion has a longer growing season and warmer summer temperatures than the rest of the province due the presence of the Grand Lake. The area is dominated by coniferous and mixed forests, but deciduous forests do occur. Trees are present in 70% of the ecoregion. Northern red oak (*Quercusrubra* L.) and bur oak are found in New Brunswick (Farrar 1995).

Ontario localities were in three ecoregions classified as 5E, 6E and 7E (Fig. 1). Six localities were in Ecoregion 5E, the Georgina Bay Ecoregion (Crins et. al 2009). Localities were mixed hardwood forests, a residential property with oak trees, and a forest edge next to a field. The climate of 5E is cool-temperate and humid. The area is dominated by mixed, deciduous, and coniferous forests typical of the Great lakes–St Lawrence Forest region. Predominant tree species are red pine (*Pinusresinosa* Sol. Ex Aiton), eastern white pine (*Pinusstrobus* L.), eastern hemlock (*Tsugacanadensis* (L.) Carrière), yellow birch (*Betulaealleghansiensis* Britt.), maple (*Acer* spp.) and oak (northern red oak, bur oak). To the south is the Lake Simcoe-Rideau Ecoregion (i.e., Ecoregion 6E) which extends from the eastern shore of Lake Huron to the Ottawa River (Crins et al. 2009). Study localities within Ecoregion 6E included a mixed hardwood forest, a park containing hardwoods, and an oak plantation. The climate is mild and moist. More than half of the ecoregion has been converted to agriculture fields. The remaining treed areas are deciduous, coniferous, and mixed forests characteristic of the Great Lakes-St Lawrence Forest region. Oak species that may be found in Ecoregion 6E include northern red oak, bur oak, black oak (*Quercusvelutina* Lam.), white oak (*Q.alba* L.), swamp white oak (*Q.bicolor* Willd.), and chinquapin oak (*Q.muelhlenbergii* Engelm.) (Farrar 1995).

Ecoregion 7E, the Lake Erie-Lake Ontario Ecoregion, is the most southerly area of Ontario (Crins et al. 2009). It incorporates shorelines from lakes Huron, Erie, and Ontario. The entire Carolinian forest region, dominated by beech, maple, black walnut, hickory, and oak forests, occurs here. This area is considered unique to Canada because it is the northern edge of the deciduous forest in North America, which does not occur elsewhere in Canada. The area experiences cool winters and long, hot, humid summers. Nearly 80% of the area has been converted to agriculture fields. Deciduous forests cover approximately 10% of the ecoregion. In addition to the oak species found in Ecoregion 6E, 7E may contain northern pin (*Quercusellipsoidalis* E.J. Hill), Shumard (*Q.shumardii* Buckl.), dwarf Chinquapin (*Q.prinoides* Willd.) and swamp pin oak (*Q.palustris* Muenchh.) (Farrar 1995). Ecoregion 7E is warmer than 6E and 5E and has a longer average growing season. The three ecoregions share a similar range in precipitation. Study localities were a mixed hardwood forest and two plantations in urban areas.

Flying beetles were collected using four types of traps; wind-oriented funnel traps, 12-unit Lindgren funnel traps, 12-unit modified Lindgren funnel traps, and 5-unit Synergy multitraps (Synergy Semiochemical Corp. Delta, BC). Wind-oriented funnel traps are also referred to as PVC pipe traps and wind-oriented pipe traps (Dowd et al. 1992; DiGirolomo et al. 2020). Three traps were placed at least 10 m apart at 16 localities in Ontario and Manitoba. Traps were hung from poles approximately 1.0–1.5 m in height from the ground and placed within 10 m of a red oak (*Quercusrubra*) or a bur oak (*Q.macrocarpa*). Wind-oriented traps were solely used at 14 localities and 12-unit Lindgren traps used at two localities. At four localities near Sault Ste Marie, Ontario and Fredericton, New Brunswick, five of each, wind-oriented funnel, Synergy multitrap, Lindgren funnel, and modified Lindgren funnel, were placed 20 m apart in hardwood stands containing *Quercus* species. At one additional site in New Brunswick, the same design was used except no Synergy multitraps were deployed. These traps were also hung from poles, at the same height from the ground.

The wind-oriented funnel traps (Dowd et al. 1992) included a collection chamber with insecticidal strips (19.2% Diclovos, “Ortho Home Defence Max No-Pest Insecticide Strip”) and a lure chamber containing fermenting flour dough plus commercial lure for Carpophilus (Megacarpolus) sayi Parsons, 1943 and *Colopterustruncatus* (Randall, 1938) beetles (Great Lakes IPM, Vestaburg MI) (Jagemann et al. 2018). The yeast dough was replaced every week, the pheromone lures every four weeks, and the insecticidal strips each month. Lindgren-funnel traps (Lindgren 1983), modified Lindgren traps (Miller et al. 2013), and Synergy multitraps were set up and maintained with the same lures and schedule as the wind-oriented funnel traps. Lures and baits were hung inside the top funnel for modified traps and outside for non-modified traps (Miller et al. 2013). The funnel cups were filled with a saltwater solution with several drops of dish detergent and replaced after every collection (Webster et al. 2012). Collections were made weekly from March to October. Samples were stored at -20 °C until they could be sorted and identified.

Specimens were initially sorted and identified by technicians (Sylvia Greifenhagen, Ontario Forest Research Institute (**OFRI**) and DD, Invasive Species Centre) and lead research scientist (SR, OFRI), later identifications were confirmed by Cucujoidea specialist (Gareth Powell, Florida State Collection of Arthropods). All specimens are deposited at OFRI in Sault Ste Marie, Ontario, except for New Brunswick collections which are held at the Atlantic Forestry Centre collection in Fredericton, NB. The generic attribution of the *Carpophilus* species was used after the papers by Kirejtshuk (2008, 2018). Information on the number of specimens recorded and collections where specimens were deposited is included in parentheses () after each record.

All species are listed with their current known distribution in Canada (Bousquet et al. 2013; Webster et al. 2016, 2022), using abbreviations from the province/territory. New records for Ontario, Manitoba are indicated in bold. The following abbreviations are used in the text and table for the used toponyms:

**AB** Alberta;

**BC** British Columbia;

**MB** Manitoba;

**NB** New Brunswick;

**NF & LB** Newfoundland & Labrador;

**NS** Nova Scotia;

**NT** Northwest Territories;

**NU** Nunavut;

**ON** Ontario;

**PE** Prince Edward Island;

**QC** Quebec;

**SK** Saskatchewan;

**YT** Yukon Territory.

Other abbreviations used in the text are:

**BG** BugGuide.net (https://bugguide.net);

**IN** iNaturalist (https://www.inaturalist.org/);

**MNRF** Ministry of Natural Resources and Forestry;

**OFRI** Ontario Forest Research Institute.

## ﻿Results

In this account, six species are reported for the first time in the province of Ontario and three species are reported for the first time in Manitoba. Reports for three species are new records for Canada (Table 1). The following are the locality data and collection dates of the new Nitidulidae records reported during this study in Ontario and Manitoba. All new species records are based on information in Bousquet et al. (2013), Webster et al. (2016, 2022), Pentinsaari et al. (2019).

**Table 1.** Distribution of *Carpophilus*, *Cychramus*, *Glischrochilus*, and *Stelidota* species reported for Canada. * = new to Canada; bold = to province, distribution from Bousquet et al. (2013) and Webster et al. (2016, 2022).

Taxa	Geographic distribution in Canada
Family Nitidulidae	
Subfamily Carpophilinae Erichson, 1842	
(Subgenus Caplothorax Kirejtshuk, 1997)	
*Carpophilusmelanopterus* Erichson, 1843	ON, QC
(Subgenus Carpophilus Stephens,1829)	
*Carpophilushemipterus* (Linnaeus, 1758)	BC, AB, MB, ON, QC, NB
(Subgenus Ecnomorphus Motchulsky, 1858)	
*Carpophilusantiquus* Melsheimer, 1844	**ON**, QC
*Carpophilusbrachypterus* (Say, 1825)	MB, ON, QC, NB, NS, PE
*Carpophiluscorticinus* Erichson, 1843*	**ON**
*Carpophilusdiscoideus* LeConte, 1858	BC, ON
(Subgenus Megacarpolus Reitter, 1919)	
*Carpophiluslugubris* Murray, 1864	BC, AB, SK, **MB**, ON
*Carpophilussayi* Parsons, 1943	SK, MB, **ON**, QC, NB, NS
(Subgenus Myothorax Murray, 1864)	
*Carpophilusdimidiatus* (Fabricius, 1792)	BC, SK, MB, ON, QC, NB
*Carpophilusmutilatus* Erichson, 1843	BC
*Carpophilusnepos* Murray, 1864*	**MB**, **ON**
(Subgenus Semocarpolus Kirejtshuk, 2008)	
*Carpophilusmarginellus* Motschulsky, 1858	SK, MB, ON, QC, NB, NS, PE
Subfamily Nitidulinae Latreille, 1802	
Tribe Cychramini Gistel, 1848	
*Cychramusadustus* Erichson, 1843	**MB**, ON, QC, NB
Tribe Nitidulini Latreille, 1802	
*Stelidotacoenosa* Erichson, 1843	**ON**, NB
*Stelidotageminata* (Say, 1825)	ON, QC
*Stelidotaoctomaculata* (Say, 1825)	ON, QC, NB, NS
Subfamily Cryptarchinae C.G. Thomson, 1859	
Tribe Cryptarchini C.G. Thomson, 1859	
(Subgenus Glischrochilus Reitter, 1873)	
*Glischrochilusconfluentus* (Say, 1823)	BC, AB, SK, MB, ON, QC, NB, NS
*Glischrochiluslecontei* W.J. Brown, 1932	BC, MB,
*Glischrochilusmoratus* W.J. Brown, 1932	BC, AB, SK, MB, ON, QC, NB, NS, PE
*Glischrochilusvittatus* (Say, 1835)	BC, AB, MB, ON, QC, NB, NS, PE
(Subgenus Librodor Reitter, 1884)	
*Glischrochilusfasciatus* (Olivier, 1790)	BC, MB, ON, QC, NB, NS, PE
*Glischrochilusobtusus* (Say, 1835)*	**ON**
*Glischrochilusquadrisignatus* (Say, 1835)	BC, AB, SK, MB, ON, QC, NB, NS, PE, NF
*Glischrochilussanguinolentussanguinolentus* (Olivier, 1790)	BC, AB, ON, QC, NB, NS, PE
*Glischrochilussiepmanni* W.J. Brown, 1932	BC, AB, SK, MB, ON, QC, NB, NS, PE

### ﻿Taxonomy

**Family Nitidulidae Latreille, 1802**

#### ﻿Subfamily Carpophilinae Erichson, 1842

##### ﻿ Carpophilus (Ecnomorphus) antiquus

Taxon classificationAnimaliaColeopteraNitidulidae

(Melsheimer, 1844)

A6555028-DD75-5420-A985-E8363258B81D

###### Notes.

This species has only been recorded in Quebec, Canada (Bousquet et al. 2013) and is prevalent across much of the eastern United States including states that border with Canada (Parsons 1943; Price and Young 2006; Morris 2020). Neither BugGuide (BG), an online entomological database hosted by Iowa State University, or iNaturalist, a citizen science database, list Ontario when reporting the distribution of the species. However, Ontario is listed under the distribution of this species in Downie and Arnett (1996) but without supporting data. Here, we recorded C. (E.) antiquus for the first time in Ontario from two localities in 2018.

###### New records.

**Ontario**: Windsor, ON, Maidstone Conservation Area off lakeshore Rd. 209, 42.2130°N, 82.7911°W, 8-v-2018 (19, OFRI); ibidem, 15-v-2018 (2, OFRI). All samples were taken from wind-oriented funnel traps, in hardwood forest next to *Quercus* sp.

London, ON, Fanshawe Conservation Area off Fanshawe Park Rd, East, 43.0507°N, 81.1818°W, 4-ix-2018, wind-oriented funnel trap, plantation forest next to *Quercus* sp. (1, OFRI).

###### Distribution in Canada.

MB, **ON**, QC (Bousquet et al. 2013).

##### ﻿ Carpophilus (Ecnomorphus) corticinus

Taxon classificationAnimaliaColeopteraNitidulidae

Erichson, 1843

D7471BC1-CDD2-5163-A735-050840D2C792

###### Notes.

This is a new record for Canada and Ontario. Carpophilus (Ecnomorphus) corticinus was collected in four different localities across the Southern region of Ontario in 2018, 2019, and 2020. There are no other records of this species for Canada in either BN or IN. However, Ontario is listed under the distribution description for Price’s (2003) thesis of Kateretidae and Nitidulidae of Wisconsin. Carpophilus (Ecnomorphus) corticinus has been recorded from US states bordering Canada, including New York, Ohio, and Michigan as well as south to Texas and Georgia (Parsons 1943; Downie and Arnett 1996).

###### New records.

**Canada, Ontario**: Windsor, ON, Maidstone Conservation Area off lakeshore Rd. 209, 42.2130°N, 82.7911°W, 17-iv-2018 (21, OFRI); ibidem, 24-iv-2018 (3, OFRI); ibidem, 8-v-2018 (7, OFRI); ibidem, 29-v-2018 (3, OFRI); ibidem, 5-vi-2018 (1, OFRI); ibidem, 19-vi-2018 (1, OFRI). All samples taken from wind-oriented funnel traps, in hardwood forest next to *Quercus* sp.

London, ON, Fanshawe Conservation Area off Fanshawe Park Rd, East, 43.0507°N, 81.1818°W, 29-v-2018 wind-oriented funnel trap, plantation forest next to *Quercus* sp. (4, OFRI).

Hamilton, ON, Royal Botanical Gardens, off Homestead Ave, 43.2882°N, 79.9069°W, 8-v-2019 (4, OFRI); ibidem, 22-v-2019 (1, OFRI); ibidem, 29-v-2019 (2, OFRI); ibidem, 12-vi-2019 (1, OFRI); ibidem, 19-vi-2019 (5, OFRI); ibidem, 3-vii-2019 (2, OFRI); ibidem, 24-vii-2019 (1, OFRI) ; ibidem, 31-vii-2019 (1, OFRI); ibidem, 14-viii-2019 (1, OFRI). All samples taken from wind-oriented funnel traps, in planted hardwood forest next to *Quercus* sp.

London, ON, Fanshawe Conservation Area off Fanshawe Park Rd, East, 43.0507°N, 81.1818°W, 10-iv-2019 (1, OFRI); ibidem, 24-iv-2019 (1, OFRI), note: specimen damaged; ibidem, 29-v-2019 (10, OFRI); ibidem, 5-vi-2019 (8, OFRI); ibidem, 12-vi-2019 (3, OFRI); ibidem, 19-vi-2019 (3, OFRI); ibidem, 16-vi-2019 (1, OFRI); ibidem, 17-vii-2019 (1, OFRI); ibidem, 18-ix-2019 (1, OFRI); ibidem, 25-ix-2019 (1, OFRI); ibidem, 2-x-2019 (1, OFRI); ibidem, 16-x-2019 (1, OFRI). All samples taken from wind-oriented funnel traps, in plantation forest next to *Quercus* sp.

Hamilton, ON, Royal Botanical Gardens, off Homestead Ave, 43.2882°N, 79.9069°W, 28-v-2020 (3, OFRI); ibidem, 3-vi-2020 (4, OFRI); ibidem, 11-vi-2020 (4, OFRI); ibidem, 18-vi-2020 (4, OFRI); ibidem, 25-vi-2020 (1, OFRI); ibidem, 8-vii-2020 (2, OFRI); ibidem, 16-vii-2020 (1, OFRI); ibidem, 30-vii-2020 (1, OFRI); ibidem, 26-viii-2020 (1, OFRI); ibidem, 17-ix-2020 (3, OFRI). All samples taken from wind-oriented funnel traps, in planted hardwood forest next to *Quercus* sp.

London, ON, Fanshawe Conservation Area off Fanshawe Park Rd, East, 43.0507°N, 81.1818°W, 27-v-2020 (13, OFRI); ibidem, 10-vi-2020 (7, OFRI); ibidem, 17-vi-2020 (4, OFRI); ibidem, 24-vi-2020 (2, OFRI); ibidem, 8-vii-2020 (1, OFRI); ibidem, 15-vii-2020 (3, OFRI); ibidem, 12-viii-2020 (2, OFRI); ibidem, 28-x-2020 (1, OFRI). All samples taken from wind-oriented funnel traps, in plantation forest next to *Quercus* sp.

Guelph, ON, The Arboretum, University of Guelph, 43.5436°N, 80.2205°W, 25-vi-2020 (1, OFRI); ibidem, 8-vii-2020 (1, OFRI); ibidem, 3-ix-2020 (1, OFRI). All samples taken from wind-oriented funnel traps, in *Quercusrubra* plantation.

###### Distribution in Canada.

**ON** (new Canadian record).

##### ﻿ Carpophilus (Megacarpolus) lugubris

Taxon classificationAnimaliaColeopteraNitidulidae

Murray, 1864

927E9DFC-6801-59F0-AD1C-8E2D607409C7

###### Notes.

This species is recorded for much of central and western Canada from British Columbia to Saskatchewan and Ontario (Bousquet et al. 2013). Here, we report Carpophilus (Megacarpolus) lugubris for the first time in Manitoba from three localities in 2019, 2020, and 2021. This species being found in Manitoba was not unexpected since it is present in the bordering provinces of Saskatchewan and Ontario. Downie and Arnett (1996) record C. (M.) lugubris occurring across the United States. There are several records of this species in Canada reported on the IN website, from BC, ON, and QC (Lugubris 2023).

###### New records.

**Manitoba**: Birds Hill Provincial Park, MB, Roscoe Rd. 50.0436°N, 96.8719°W, 10-ix-2019 wind-oriented funnel trap, mixed hardwood forest next to *Quercus* sp. (1, OFRI).

Beaudry Provincial Park, MB, Roblin Blvd, 49.8576°N, 97.4638°W, 11-viii-2020 (1, OFRI), ibidem, 18-viii-2020 (1, OFRI); ibidem, 2-ix-2020 (2, OFRI); ibidem, 23-ix-2020 (1, OFRI); ibidem, 30-ix-2020 (1, OFRI). All samples were collected from wind-oriented funnel traps, riparian hardwood forest next to *Quercus* sp.

Saint Malo, St Malo Provincial Park, MB, 49.3229°N, 96.9355°W, 29-vii-2021, wind-oriented funnel trap, mixed hardwood forest next to *Quercus* sp. (1, OFRI).

###### Distribution in Canada.

BC, AB, SK, **MB**, ON (Bousquet et al. 2013).

##### ﻿ Carpophilus (Myothorax) nepos

Taxon classificationAnimaliaColeopteraNitidulidae

Murray, 1864

006E153A-C236-57EF-8539-8F7160216329

###### Notes.

This is the first record for Carpophilus (Myothorax) nepos in Canada, obtained from five localities in Ontario in 2018, 2019, and 2020 and four localities in Manitoba in 2019 and 2021. This species, associated with stored grain products, is almost cosmopolitan and its distribution is poorly understood due to its synonym with *Carpophilusfreemani* Dobson, 1956 (Kirejtshuk 1996). Peck and Thomas (1998) mention that this species has been recorded in the United States and Price (2003) provides records for the state of Wisconsin. Carpophilus (Myothorax) nepos has not been recorded in Canada according to Bousquet et al. (2013).

###### New records.

**Canada, Ontario**: Windsor, ON, Maidstone Conservation Area off lakeshore Rd. 209, 42.2130°N, 82.7911°W, 15-v-2018, wind-oriented funnel trap, hardwood forest next to *Quercus* sp. (1, OFRI).

Ottawa, ON, Elmhurst Park, off Alpine Ave, 45.3591°N, 75.7861°W, 23-viii-2019, wind-oriented funnel trap, in a hardwood forest next to *Quercus* sp. (2, OFRI).

Hamilton, ON, Royal Botanical Gardens, off Homestead Ave, 43.2882°N, 79.9069°W, 3-vii-2019 (1, OFRI); ibidem, 18-ix-2019 (1, OFRI); ibidem, 2-x-2019 (2, OFRI). All samples taken from wind-oriented funnel traps, in a planted hardwood forest, next to *Quercus* sp.

London, ON, Fanshawe Conservation Area off Fanshawe Park Rd, East, 43.0507°N, 81.1818°W, 4-ix-2019 (1, OFRI); ibidem, 11-ix-2019 (1, OFRI); ibidem, 24-ix-2019 (1, OFRI). All samples taken from wind-oriented funnel traps, in a plantation forest, next to *Quercus* sp.

Guelph, ON, The Arboretum, University of Guelph, 43.5436°N, 80.2205°W, 3-vii-2019 (1, OFRI); ibidem, 43.5436°N, 80.2205°W, 18-ix-2019 (1, OFRI). All samples taken from wind-oriented funnel traps in a *Quercusrubra* plantation.

Ottawa, ON, Elmhurst Park, off Alpine Ave, 45.3591°N, 75.7861°W, 27-viii-2020 wind-oriented funnel trap in hardwood forest next to *Quercus* sp. (1, OFRI).

Hamilton, ON, Royal Botanical Gardens, off Homestead Ave, 43.2882°N, 79.9069°W, 30-vii-2020 (1, OFRI); ibidem, 5-viii-2020 (1, OFRI); ibidem, 12-viii-2020 (1, OFRI); ibidem, 17-ix-2020 (1, OFRI). All samples taken from wind-oriented funnel traps, in a planted hardwood forest, next to *Quercus* sp.

London, ON, Fanshawe Conservation Area off Fanshawe Park Rd, East, 43.0507°N, 81.1818°W, 15-vii-2020 (2, OFRI); ibidem, 22-vii-2020 (2, OFRI); ibidem, 28-x-2020 (1, OFRI). All samples taken from wind-oriented funnel traps, in a plantation forest, next to *Quercus* sp.

Guelph, ON, The Arboretum, University of Guelph, 43.5436°N, 80.2205°W, 12-vii-2020 (4, OFRI); ibidem, 10-ix-2020 (2, OFRI); ibidem, 24-ix-2020 (3, OFRI). All samples taken from wind-oriented funnel traps, in a *Quercusrubra* plantation.

###### New records.

**Canada, Manitoba**: Birds Hill Provincial Park, MB, Roscoe Rd. 50.0436°N, 96.8719°W, 2-vii-2019, wind-oriented funnel trap, mixed hardwood forest next to *Quercus* sp. (2, OFRI).

Beaudry Provincial Park, MB, Roblin Blvd, 49.8576°N, 97.4638°W, 11-vi-2019, wind-oriented funnel trap, riparian hardwood forest next to *Quercus* sp. (2, OFRI).

Winnipeg, MB, Winnipeg James Armstrong Richardson International Airport off Wihuri Rd., 49.8996°N, 97.2538°W, 11-vi-2019 (1, OFRI); ibidem, 25-vi-2019 (1, OFRI); ibidem, 14-viii-2019 (2, OFRI); ibidem, 22-viii-2019 (1, OFRI). All samples taken from wind-oriented funnel traps, in a mixed hardwood forest next to *Quercus* sp.

Saint Malo, MB, St Malo Provincial Park, 49.3229°N, 96.9355°W, 3-vi-2021, wind-oriented funnel trap, mixed hardwood forest next to *Quercus* sp. (1, OFRI).

###### Distribution in Canada.

**ON**, **MB** (new Canadian record).

##### ﻿ Carpophilus (Megacarpolus) sayi

Taxon classificationAnimaliaColeopteraNitidulidae

Parsons, 1943

E4F2B614-DD99-501A-93D0-34202C1264AC

###### Notes.

Here, we report Carpophilus (Megacarpolus) sayi for the first time in Ontario from 12 localities in 2018, 2019, 2020, and 2021. The species is found throughout central Canada excluding Ontario (Bousquet et al. 2013) and along the northern United States bordering Canada (Downie and Arnett 1996). These records suggest that this species could be found throughout Ontario. BugGuide does not specifically exclude Ontario from its distribution; however, Downie and Arnett (1996) include Ontario in their distribution description. iNaturalist does include one locality of C. (M.) sayi near Kitchener, Ontario (Jakubowski 2021) with a photo attached. The photo submitted to IN could be Carpophilus (Megacarpolus) sayi or C. (M.) lugubris. The two species sometimes look similar enough that Parsons (1943) suggested they could hybridize. According to Parsons (1943) the males of C. (M.) lugubris have two, deep, circular depressions on the hypopygidium and females have a pygidium that is blunt carinate, shining, and tuberculiform at the apex. In contrast, the males of C. (M.) sayi have two, large, vague, shallow depression on the hind margin of their hypopygidium and females have a distinct, blunt carina on the pygidium (Parsons 1943). Price (2003) also points out that C. (M.) sayi has rectangular humeral angles while the elytral humerals of C. (M.) lugubris are rounded or obtuse.

###### New records.

**Ontario**: Sault Ste. Marie, ON, Wattswood Park and Ski Trails off Mt. Pleasant Ct., 46.3356°N, 84.2527°W, 14-v-2018 (10, OFRI); ibidem, 20-v-2018 (11, OFRI); ibidem, 27-v-2018 (123, OFRI); ibidem,, 6-vi-2018 (31, OFRI); ibidem, 10-vi-2018 (4, OFRI); ibidem, 25-vi-2018 (11, OFRI); ibidem, 1-vii-2018 (1, OFRI); ibidem, 4-vii-2018 (3, OFRI); ibidem, 11-vii-2018 (1, OFRI); ibidem, 18-vii-2018 (2, OFRI); ibidem, 28-vii-2018 (15, OFRI). All samples were taken from wind-oriented funnel traps, in a mixed hardwood forest, next to *Quercus* sp.

Peterborough, ON Northumberland County Forest off Dunbar Rd., 17-v-2018 (5, OFRI); ibidem, 24-v-2018 (15, OFRI); ibidem, 31-v-2018 (11, OFRI); ibidem, 14-vi-2018 (20, OFRI); ibidem, 19-vi-2018 (18, OFRI); ibidem, 29-vi-2018 (6, OFRI); ibidem, 6-vii-2018 (25, OFRI); ibidem, 13-vii-2018 (4, OFRI); ibidem, 19-vii-2018 (1, OFRI); ibidem, 26-vii-2018 (4, OFRI). All samples were taken from wind-oriented funnel traps, in a mixed hardwood forest, next to *Quercus* sp.

Sault Ste. Marie, ON, Hiawatha Highlands off Fish Hatchery Rd., 46.3436°N, 84.1705°W, 15-v-2019 (2, OFRI); ibidem, 29-v-2019 (28, OFRI); ibidem, 5-vi-2019 (7, OFRI); ibidem, 12-vi-2019 (90, OFRI); ibidem, 19-vi-2019 (16, OFRI); ibidem, 26-vi-2019 (10, OFRI); ibidem, 3-vii-2019 (4, OFRI); ibidem, 9-vii-2019 (1, OFRI); ibidem, 17-vii-2019 (6, OFRI); ibidem, 31-vii-2019 (4, OFRI); ibidem, 8-viii-2019 (4, OFRI); ibidem, 14-viii-2019 (16, OFRI); ibidem, 21-viii-2019 (3, OFRI); ibidem, 28-viii-2019 (2, OFRI). All samples were taken from wind-oriented funnel traps, in mixed hardwood forest, next to *Quercus* sp.

Ottawa, ON, Elmhurst Park, off Alpine Ave, 45.3591°N, 75.7861°W, 8-v-2019 (1, OFRI); ibidem, 12-vi-2019 (1, OFRI); ibidem, 25-vi-2019 (1, OFRI); ibidem, 31-vii-2019 (1, OFRI); ibidem, 7-viii-2019 (1, OFRI). All samples were taken from wind-oriented funnel traps, in a hardwood forest, next to *Quercus* sp.

Hamilton, ON, Royal Botanical Gardens, off Homestead Ave, 43.2882°N, 79.9069°W, 22-v-2019 (3, OFRI); ibidem, 29-v-2019 (61, OFRI); ibidem, 5-vi-2019 (6, OFRI); ibidem, 12-vi-2019 (3, OFRI); ibidem, 19-vi-2019 (12, OFRI); ibidem, 26-vi-2019 (1, OFRI); ibidem, 3-vii-2019 (5, OFRI); ibidem, 10-vii-2019 (1, OFRI). All samples were taken from wind-oriented funnel traps, in a planted hardwood forest, next to *Quercus* sp.

North Bay, ON, Canadore College trails, off College Dr., 46.3427°N, 79.5033°W, 30-v-2019 (15, OFRI); ibidem, 6-vi-2019 (5, OFRI); ibidem, 13-vi-2019 (70, OFRI); ibidem, 20-vi-2019 (10, OFRI); ibidem, 27-vi-2019 (10, OFRI); ibidem, 4-vii-2019 (8, OFRI); ibidem, 11-vii-2019 (3, OFRI); ibidem, 18-vii-2019 (5, OFRI); ibidem, 25-vii-2019 (1, OFRI); ibidem, 12-ix-2019 (1, OFRI). All samples were taken from wind-oriented funnel traps, in a mixed hardwood forest, next to *Quercus* sp.

London, ON, Fanshawe Conservation Area off Fanshawe Park Rd, East, 43.0507°N, 81.1818°W, 15-vi-2019 (1, OFRI); ibidem, 26-vi-2019 (1, OFRI). Both samples were taken from wind-oriented funnel traps, in a mixed hardwood forest, next to *Quercus* sp.

Guelph, ON, The Arboretum, University of Guelph, 43.5436°N, 80.2205°W, 5-vi-2019 (2, OFRI); ibidem, 12-vi-2019 (2, OFRI); ibidem, 19-vi-2019 (4, OFRI). All samples were taken from wind-oriented funnel traps, in a *Quercusrubra* plantation.

Sault Ste. Marie, ON, Hiawatha Highlands off Fish Hatchery Rd., 46.3436°N, 84.1705°W, 19-v-2020 (18, OFRI); ibidem, 20-v-2020 (14, OFRI); ibidem, 25-v-2020 (18, OFRI); ibidem, 27-v-2020 (8, OFRI); ibidem, 1-vi-2020 (28, OFRI); ibidem, 2-vi-2020 (8, OFRI); ibidem, 3-vi-2020 (3, OFRI); ibidem, 4-vi-2020 (6, OFRI); ibidem, 5-vi-2020 (3, OFRI); ibidem, 8-vi-2020 (5, OFRI); ibidem, 10-vi-2020 (6, OFRI); ibidem, 14-vi-2020 (2, OFRI); ibidem, 15-vi-2020 (3, OFRI); ibidem, 16-vi-2020 (8, OFRI); ibidem, 17-vi-2020 (2, OFRI); ibidem, 25-vi-2020 (3, OFRI); ibidem, 26-vi-2020 (1, OFRI); ibidem, 3--vi-2020 (1, OFRI); ibidem, 8-vii-2020 (3, OFRI); ibidem, 15-vii-2020 (9, OFRI); ibidem, 22-vii-2020 (11, OFRI); ibidem, 29-vii-2020 (7, OFRI); ibidem, 5-viii-2020 (13, OFRI); ibidem, 12-viii-2020 (17, OFRI); ibidem, 19-viii-2020 (22, OFRI); ibidem, 26-viii-2020 (9, OFRI); ibidem, 2-ix-2020 (2, OFRI); ibidem, 9-ix-2020 (3, OFRI); ibidem, 17-ix-2020 (1, OFRI); Sault Ste. Marie, ON, Hiawatha Highlands off Fish Hatchery Rd., 46.5601°N, 84.4193°W, 19-v-2020 (3, OFRI). Almost all samples were taken from the wind-oriented funnel trap, in a mixed hardwood forest, next to *Quercus* sp., but the sample of 19-v-2020 was collected from the Lindgren funnel trap, in a mixed regenerated forest, next to *Quercus* sp.

Sault Ste. Marie, ON, Maki Rd., 46.5601°N, 84.4193°W, 18-v-2020 (1, OFRI); ibidem, 20-v-2020 (1, OFRI); ibidem, 21-v-2020 (24, OFRI); ibidem, 22-v-2020 (34, OFRI); ibidem, 23-v-2020 (20, OFRI); ibidem, 24-v-2020 (15, OFRI); ibidem, 25-v-2020 (17, OFRI); ibidem, 27-v-2020 (10, OFRI); ibidem, 28-v-2020 (6, OFRI); ibidem, 2-vi-2020 (1, OFRI); ibidem, 3-vi-2020 (3, OFRI); ibidem, 4-vi-2020 (5, OFRI); ibidem, 8-vi-2020 (8, OFRI); ibidem, 9-vi-2020 (12, OFRI); ibidem, 10-vi-2020 (4, OFRI); ibidem, 14-vi-2020 (4, OFRI); ibidem, 15-vi-2020 (5, OFRI); ibidem, 16-vi-2020 (4, OFRI); ibidem, 17-vi-2020 (1, OFRI); ibidem, 19-vi-2020 (3, OFRI); ibidem, 20-vi-2020 (1, OFRI); ibidem, 21-vi-2020 (1, OFRI); ibidem, 8-vii-2020 (8, OFRI); ibidem, 15-vii-2020 (2, OFRI); ibidem, 21-vii-2020 (2, OFRI); ibidem, 29-vii-2020 (4, OFRI); ibidem, 5-viii-2020 (1, OFRI); ibidem, 12-viii-2020 (6, OFRI); ibidem, 18-viii-2020 (1, OFRI); ibidem, 2-ix-2020 (1, OFRI); ibidem, 16-ix-2020 (1, OFRI); ibidem, 30-ix-2020 (1, OFRI). All samples were taken from Lindgren funnel traps, in a mixed regenerated forest, next to *Quercus* sp.

Sault Ste. Marie, ON, Retta St., 46.5061°N, 84.2945°W, 24-v-2020 (2, OFRI); ibidem, 25-v-2020 (12, OFRI); ibidem, 27-v-2020 (10, OFRI); ibidem, 1-vi-2020 (14, OFRI); ibidem, 2-vi-2020 (9, OFRI); ibidem, 3-vi-2020 (16, OFRI); ibidem, 4-vi-2020 (11, OFRI); ibidem, 5-vi-2020 (5, OFRI); ibidem, 8-vi-2020 (14, OFRI); ibidem, 9-vi-2020 (26, OFRI); ibidem, 10-vi-2020 (8, OFRI); ibidem, 16-vi-2020 (3, OFRI); ibidem, 17-vi-2020 (4, OFRI); ibidem, 18-vi-2020 (2, OFRI); ibidem, 20-vi-2020 (1, OFRI); ibidem, 1-vii-2020 (9, OFRI); ibidem, 8-vii-2020 (34, OFRI); ibidem, 15-vii-2020 (8, OFRI); ibidem, 22-vii-2020 (12, OFRI); ibidem, 29-vii-2020 (9, OFRI); ibidem, 5-viii-2020 (3, OFRI); ibidem, 19-viii-2020 (2, OFRI); ibidem, 12-viii-2020 (4, OFRI); ibidem, 30-ix-2020 (1, OFRI). All samples were taken from Lindgren funnel traps, among residential hardwood trees (*Quercus* sp.).

Ottawa, ON, Elmhurst Park, off Alpine Ave, 45.3591°N, 75.7861°W, 29-v-2020 (9, OFRI); ibidem, 4-v-2020 (1, OFRI); ibidem, 11-v-2020 (17, OFRI); ibidem, 19-v-2020 (1, OFRI); ibidem, 24-v-2020 (1, OFRI); ibidem, 10-vii-2020 (2, OFRI); ibidem, 24-vii-2020 (1, OFRI). All samples were taken from wind-oriented funnel traps, in a hardwood forest, next to *Quercus* sp.

Hamilton, ON, Royal Botanical Gardens, off Homestead Ave, 43.2882°N, 79.9069°W, 28-v-2020 (21, OFRI); ibidem, 3-vi-2020 (48, OFRI); ibidem, 11-vi-2020 (58, OFRI); ibidem, 18-vi-2020 (31, OFRI); ibidem, 25-vi-2020 (8, OFRI); ibidem, 2-vii-2020 (11, OFRI); ibidem, 8-vii-2020 (2, OFRI); ibidem, 16-vii-2020 (14, OFRI); ibidem, 23-vii-2020 (6, OFRI); ibidem, 30-vii-2020 (3, OFRI); ibidem, 5-viii-2020 (8, OFRI); ibidem, 12-viii-2020 (1, OFRI); ibidem, 26-viii-2020 (1, OFRI); ibidem, 17-ix-2020 (1, OFRI). All samples were taken from wind-oriented funnel traps, in a planted hardwood forest, next to *Quercus* sp.

North Bay, ON, Canadore College trails, off College Dr., 46.3427°N, 79.5033°W, 20-v-2020 (3, OFRI); ibidem, 27-v-2020 (193, OFRI); ibidem, 3-vi-2020 (3, OFRI); ibidem, 10-vi-2020 (34, OFRI); ibidem, 17-vi-2020 (9, OFRI); ibidem, 24-vi-2020 (6, OFRI); ibidem, 1-vii-2020 (14, OFRI); ibidem, 8-vii-2020 (15, OFRI); ibidem, 15-vii-2020 (1, OFRI); ibidem 29-vii-2020 (7, OFRI). ; ibidem, 5-viii-2020 (2, OFRI). All samples were taken from wind-oriented funnel traps, in a mixed hardwood forest, next to *Quercus* sp.

London, ON, Fanshawe Conservation Area, 43.0507°N, 81.1818°W, 24-vi-2020 wind-oriented funnel trap, plantation forest, next to *Quercus* sp. (1, OFRI).

Guelph, ON, The Arboretum, University of Guelph, 43.5436°N, 80.2205°W, 3-vi-2020; ibidem, 11-vi-2020 (1, OFRI); ibidem, 16-vii-2020 (1, OFRI); ibidem, 30-vii-2020 (1, OFRI). All samples were taken from wind-oriented funnel traps, in a *Quercusrubra* plantation.

Sault Ste. Marie, Goulais Ave, Crimson Ridge golf course, 46.5767°N, 84.3799°W, 18-v-2021, Lindgren funnel (71, OFRI), modified Lindgren funnel (40, OFRI), synergy multitrap (120, OFRI), wind-oriented funnel trap (55, OFRI); ibidem,, 25-v-2021, Lindgren funnel (159, OFRI), modified Lindgren funnel (59, OFRI), synergy multitrap (200, OFRI), wind-oriented funnel trap (73, OFRI); ibidem,46.5767°N, 84.3799°W, 1-vi-2021, Lindgren funnel (166, OFRI), modified Lindgren funnel (35, OFRI), synergy multitrap (75, OFRI), wind-oriented funnel trap (21, OFRI); ibidem, 8-vi-2021, Lindgren funnel (162, OFRI), modified Lindgren funnel (81, OFRI), synergy multitrap (155, OFRI), wind-oriented funnel trap (35, OFRI); ibidem, 15-vi-2021, Lindgren funnel (34, OFRI), modified Lindgren funnel (18, OFRI), synergy multitrap (25, OFRI), wind-oriented funnel trap (3, OFRI); ibidem, 22-vi-2021, Lindgren funnel (25, OFRI), modified Lindgren funnel (16, OFRI), synergy multitrap (37, OFRI), wind-oriented funnel trap (6, OFRI); ibidem, 29-vi-2021, Lindgren funnel (7, OFRI), modified Lindgren funnel (4, OFRI), synergy multitrap (8, OFRI), wind-oriented funnel trap (7, OFRI); ibidem, 6-vii-2021, Lindgren funnel (5, OFRI), modified Lindgren funnel (4, OFRI), wind-oriented funnel trap (7, OFRI); ibidem, 13-vii-2021, Lindgren funnel (8, OFRI), modified Lindgren funnel (8, OFRI), synergy multitrap (8, OFRI), wind-oriented funnel trap (6, OFRI); ibidem, 20-vii-2021, Lindgren funnel (16, OFRI), modified Lindgren funnel (2, OFRI), synergy multitrap (7, OFRI), wind-oriented funnel trap (3, OFRI); ibidem, 27-vii-2021, Lindgren funnel (9, OFRI), modified Lindgren funnel (3, OFRI), synergy multitrap (5, OFRI), wind-oriented funnel trap (1, OFRI); ibidem, 3-viii-2021, modified Lindgren funnel (2, OFRI); ibidem, 10-viii-2021, Lindgren funnel (2, OFRI), synergy multitrap (1, OFRI); ibidem, 17-viii-2021, synergy multitrap (1, OFRI), wind-oriented funnel trap (1, OFRI); ibidem, 22-ix-2021, synergy multitrap (1, OFRI). All samples were collected from a mixed hardwood forest, next to *Quercus* sp.

Sault Ste. Marie, ON, Landslide Rd., 46.5792°N, 84.2802°W, 18-v-2021, Lindgren funnel trap (340, OFRI), modified Lindgren funnel trap (183, OFRI), synergy multitrap (468, OFRI), wind-oriented funnel trap (340, OFRI); ibidem, 18-v-2021, Lindgren funnel (276, OFRI), modified Lindgren funnel (160, OFRI), synergy multitrap (299, OFRI), wind-oriented funnel trap (174, OFRI); ibidem, 1-vi-2021, Lindgren funnel (151, OFRI), modified Lindgren funnel (77, OFRI), synergy multitrap (210, OFRI), wind-oriented funnel trap (33, OFRI); ibidem, 8-vi-2021, Lindgren funnel (148, OFRI), modified Lindgren funnel (198, OFRI), synergy multitrap (277, OFRI), wind-oriented funnel trap (54, OFRI ; ibidem, 15-vi-2021, Lindgren funnel (69, OFRI), modified Lindgren funnel (27, OFRI), synergy multitrap (67, OFRI), wind-oriented funnel trap (15, OFRI); ibidem, 22-v-2021, Lindgren funnel (23, OFRI), modified Lindgren funnel (18, OFRI), synergy multitrap (59, OFRI),wind-oriented funnel trap (17, OFRI); ibidem, 29-vi-2021, Lindgren funnel (22, OFRI), modified Lindgren funnel (23, OFRI), synergy multitrap (29, OFRI), wind-oriented funnel trap (12, OFRI); ibidem, 6-vii-2021, Lindgren funnel (12, OFRI), modified Lindgren funnel (15, OFRI), synergy multitrap (30, OFRI), wind-oriented funnel trap (6, OFRI); ibidem, 13-vii-2021, Lindgren funnel (9, OFRI), modified Lindgren funnel (20, OFRI), synergy multitrap (29, OFRI), wind-oriented funnel trap (10, OFRI); ibidem, 20-vii-2021, Lindgren funnel (17, OFRI), modified Lindgren funnel (18, OFRI), synergy multitrap (28, OFRI), wind-oriented funnel trap (5, OFRI; ibidem, 27-vii-2021, Lindgren funnel (4, OFRI), modified Lindgren funnel (10, OFRI), synergy multitrap (10, OFRI), wind-oriented funnel trap (4, OFRI); ibidem, 3-viii-2021, Lindgren funnel (1, OFRI), modified Lindgren funnel (2, OFRI), synergy multitrap (2, OFRI), wind-oriented funnel trap (2, OFRI); ibidem, 10-viii-2021 (1, OFRI); ibidem, 17-viii-2021 (3, OFRI); ibidem, 24-viii-2021, Lindgren funnel (1, OFRI), synergy multitrap (3, OFRI); ibidem, 7-ix-2021 (1, OFRI). All samples were collected from a mixed hardwood forest, next to *Quercus* sp.

###### Distribution in Canada.

SK, MB, **ON**, QC, NB, NS (Bousquet et al. 2013).

#### ﻿Subfamily Nitidulinae Latreille, 1802

##### Tribe Cychramini Gistel, 1848

###### ﻿ Cychramus adustus

Taxon classificationAnimaliaColeopteraNitidulidae

(Erichson, 1843)

5ADEDC12-4A4E-557A-9C1F-17120364CD17

####### Notes.

This is the first record of *Cychramusadustus* for Manitoba found in three localities in 2019. The McNamara (1991) checklist of Nitidulidae recorded *C.adustus* present in Ontario and Quebec. Majka et al. (2008) recorded *C.adustus* in New Brunswick, and Webster et al. (2022) recorded *C.adustus* in Prince Edward Island. There are five records for *C.adustus* on the IN website that are mostly collected in eastern Ontario and near Montreal, Quebec (Adustus 2023). The BN website references Bousquet et al. (2013) when describing the range of *C.adustus* as eastern North America.

####### New records.

**Manitoba**: Birds Hill Provincial Park, MB, Roscoe Rd. 50.0436°N, 96.8719°W, 14-v-2019 (4, OFRI); ibidem, 22-v-2019 (9, OFRI); ibidem, 4-vi-2019 (4, OFRI); ibidem, 11-vi-2019 (4, OFRI); ibidem, 17-vi-2019 (3, OFRI); ibidem, 25-vi-2019 (5, OFRI); ibidem, 2-vii-2019 (3, OFRI); ibidem, 31-vii-2019 (1, OFRI); ibidem, 6-viii-2019 (2, OFRI); ibidem, 28-viii-2019 (2, OFRI); ibidem, 10-ix-2019 (2, OFRI). All samples were taken from wind-oriented funnel traps, in a mixed hardwood forest, next to *Quercus* sp.

Beaudry Provincial Park, MB, Roblin Blvd, 49.8576°N, 97.4638°W, 14-v-2019 (2, OFRI); ibidem, 29-v-2019 (14, OFRI); ibidem, 4-vi-2019 (3, OFRI); ibidem, 11-vi-2019 (14, OFRI); ibidem, 25-vi-2019 (1, OFRI); ibidem, 8-vii-2019 (1, OFRI); ibidem, 16-vii-2019 (1, OFRI); ibidem, 6-viii-2019 (2, OFRI); ibidem, 28-viii-2019 (1, OFRI); ibidem, 10-ix-2019 (2, OFRI). All samples were taken from wind-oriented funnel traps, in a riparian hardwood forest, next to *Quercus* sp.

Winnipeg, MB, Winnipeg James Armstrong Richardson International Airport off Wihuri Rd., 49.8996°N, 97.2538°W, 22-v-2019 (1, OFRI); ibidem, 4-vi-2019 (4, OFRI); ibidem, 11-v-2019 (3, OFRI); ibidem, 17-vi-2019 (3, OFRI); ibidem, 25-vi-2019 (2, OFRI); ibidem, 8-vii-2019 (1, OFRI); ibidem, 31-vii-2019 (1, OFRI); ibidem, 14-viii-2019 (1, OFRI). All samples were taken from wind-oriented funnel traps, in a mixed hardwood forest, next to *Quercus* sp.

####### Distribution in Canada.

**MB**, ON, QC, NB, PE (Bousquet et al. 2013; Webster et al. 2022).

##### Tribe Nitidulini Latreille, 1802

###### ﻿ Stelidota coenosa

Taxon classificationAnimaliaColeopteraNitidulidae

Erichson, 1843

B8C68A74-B102-5FED-AE7A-1D78AAB40A74

####### Notes.

This is the first record of *Stelidotacoenosa* for Ontario. It was collected at one locality in the province in 2021. The species was first reported from boletus mushrooms in a *Pinusbanksiana* forest in Northumberland Co., New Brunswick by Webster et al. (2016). It has also been recorded from several states along the eastern United States from New York to Florida (Parsons 1943). *Stelidotacoenosa* was collected as far west as Wisconsin, south to Arizona and can be found in subtropical and tropical areas of Central and South America (Galford et al. 1991). The IN website does not have any record for *S.coenosa* in Canada while the website, BG references the report from New Brunswick (Webster et al. 2016).

####### New record.

**Ontario**: Guelph, ON, The Arboretum, University of Guelph, 43.5436°N, 80.2205°W, 5-viii-2021, wind-oriented funnel trap, in *Quercusrubra* plantation (1, OFRI).

####### Distribution in Canada.

**ON**, NB (Bousquet et al. 2013; Webster et al. 2016).

#### ﻿Subfamily Cryptarchinae C.G. Thomson, 1859

##### Tribe Cryptarchini C.G. Thomson, 1859

###### ﻿ Glischrochilus (Librodor) obtusus

Taxon classificationAnimaliaColeopteraNitidulidae

(Say, 1835)

F9243E65-6CD4-5993-B927-82EB47131BD6

####### Notes.

This 2018 collection is the first record of Glischrochilus (Librodor) obtusus in Canada and a new provincial record for Ontario. This species can be found throughout the eastern United States including the northern border states with Canada; Maine, New York, Michigan, and Wisconsin (Downie and Arnett 1996; Price and Young 2006). IN recorded locality information by Nenadov (2021) in Comber, Ontario on July 6, 2021.

####### New record.

**Ontario**: Peterborough, ON, Northumberland County Forest off Dunbar Rd., 44.0636°N, 78.0331°W, 19-vi-2018, wind-oriented funnel trap, in a mixed hardwood forest, next to *Quercus* sp. (2, OFRI).

####### Distribution in Canada.

**ON** (New Canadian record).

## ﻿Discussion

In 2013, Bousquet et al. (2013) reported 99 species of Nitidulidae from Canada. This included 45 species from Manitoba and 63 from Ontario. In addition, three Palaearctic species were recently recorded in Canada, the first one from New Brunswick (Webster et al. 2016), the second one from Prince Edward Island (Webster et al. 2022), and the third one from Ontario (Pentinsaari et al. 2019). Brunke et al. (2019) suggested that the total number of Nitidulidae species in Canada is even higher. They used an analysis of BOLD barcode index numbers to estimate that as many as 12 Nitidulidae species from Canada have not been described or reported. Here, we report six new species for Ontario, Carpophilus (Megacarpolus) sayi, C. (Ecnomorphus) antiquus, C. (E.) corticinus, C. (Myothorax) nepos, Glischrochilus (Librodor) obtusus, and *Stelidotacoenosa* bringing the total to 70, and three species for Manitoba, Carpophilus (Megacarpolus) lugubris, C. (M.) nepos, and *Cychramusadustus*, bringing the total to 48. Three of these reports, Carpophilus (Ecnomorphus) corticinus, C. (Myothorax) nepos, and *Glischrochilusobtusus* are new reports for Canada bringing the total to 104 or 105 depending on the inclusion of Cybocephalinae as a subfamily in the family of Nitidulidae (Kirejtshuk 2008; Kirejtshuk and Mantič 2015) or consideration of this group as a separate family (Cline et al. 2014; Smith 2022).

The new records fill gaps in our knowledge of these species’ distributions. Of the new species found in Ontario, Carpophilus (Megacarpolus) sayi was the most frequently reported species with records at 13 of 14 survey localities. Carpophilus (Myothorax) nepos also appears widespread, being reported at numerous localities throughout southern Ontario (i.e., ecoregions 6 and 7) and in Manitoba.

Four new records occurred only in southern Ontario (i.e., ecoregions 6E, 7E), indicating that more surveys in this region could increase our knowledge of Nitidulidae diversity in Canada. One reason may be that part of this region contains the northernmost extent of the deciduous forest type, sometimes called the Carolinian Forest Region or the Deciduous Forest Region (Crins et al. 2009). The diversity of flora and fauna is greater here than in other parts of Canada. This region is warmer and has a longer growing season than the rest of Ontario because of its proximity to three large lakes and its southern position (Crins et al. 2009). In addition, further surveying in Manitoba is needed to clarify if Carpophilus (Megacarpolus) lugubris, C. (Myothorax) nepos, and *Cychramusadustus* occur outside the greater Winnipeg area. No new records were made for New Brunswick, suggesting that recent efforts to more fully describe the beetle diversity of this province have been reasonably complete (Majka et al. 2008; Webster et al. 2016).

The new species recorded in this study have been collected from fungi or sap flows and some are known to transmit fungal spores and fragments of mycelia (Price 2003). New detections of mycetophagous beetles in Canada are important, further elucidating how fungi and fungal diseases are spread across Canada’s treed landscapes. Especially species of *Carpophilus*, *Glischrochilus*, *Cychramus*, *Stelidota*, and others that have been found at oak wilt mats or other fungal structures (Price 2003).

## Supplementary Material

XML Treatment for Carpophilus (Ecnomorphus) antiquus

XML Treatment for Carpophilus (Ecnomorphus) corticinus

XML Treatment for Carpophilus (Megacarpolus) lugubris

XML Treatment for Carpophilus (Myothorax) nepos

XML Treatment for Carpophilus (Megacarpolus) sayi

XML Treatment for Cychramus adustus

XML Treatment for Stelidota coenosa

XML Treatment for Glischrochilus (Librodor) obtusus
